# Ligands as Stabilizers of G-Quadruplexes in Non-Coding RNAs

**DOI:** 10.3390/molecules26206164

**Published:** 2021-10-13

**Authors:** Joana Figueiredo, Tiago Santos, André Miranda, Daniela Alexandre, Bernardo Teixeira, Pedro Simões, Jéssica Lopes-Nunes, Carla Cruz

**Affiliations:** CICS-UBI—Centro de Investigação em Ciências da Saúde, Universidade da Beira Interior, Av. Infante D. Hen-rique, 6200-506 Covilhã, Portugal; figueiredo_joana@hotmail.com (J.F.); tiagoaasantos@hotmail.com (T.S.); andre.miranda@ubi.pt (A.M.); danilex.ct3@gmail.com (D.A.); b.teixeira6200@gmail.com (B.T.); pedro.miguel.baltazar.simoes@ubi.pt (P.S.); jessicalonu@hotmail.com (J.L.-N.)

**Keywords:** non-coding RNAs, miRNA biogenesis, RNA G-quadruplex, ligands

## Abstract

The non-coding RNAs (ncRNA) are RNA transcripts with different sizes, structures and biological functions that do not encode functional proteins. RNA G-quadruplexes (rG4s) have been found in small and long ncRNAs. The existence of an equilibrium between rG4 and stem−loop structures in ncRNAs and its effect on biological processes remains unexplored. For example, deviation from the stem−loop leads to deregulated mature miRNA levels, demonstrating that miRNA biogenesis can be modulated by ions or small molecules. In light of this, we report several examples of rG4s in certain types of ncRNAs, and the implications of G4 stabilization using small molecules, also known as G4 ligands, in the regulation of gene expression, miRNA biogenesis, and miRNA−mRNA interactions. Until now, different G4 ligands scaffolds were synthesized for these targets. The regulatory role of the above-mentioned rG4s in ncRNAs can be used as novel therapeutic approaches for adjusting miRNA levels.

## 1. Introduction

**Figure 1:**
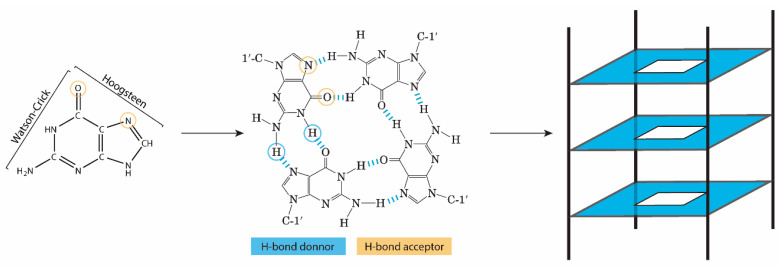
Illustration of G-tetrad formation through Hoogsteen bonds and G-tetrads stacking.

The ncRNAs are RNAs that are not translated into proteins and are divided into two main categories based on their size: small ncRNAs with size < 200 nt (e.g., miRNA, piRNA, and sRNAs) and long ncRNAs with size ≥ 200 nt (e.g., lincRNA and NAT) [1]. ncRNAs can fold into complex structures and interact with proteins, DNA and other RNAs, modulating the activity, DNA targets, or partners of multiprotein complexes [2]. Thus, ncRNAs can be regulators of different diseases (cancer, cardiovascular and neurological disorders) and can open new avenues to novel therapeutic approaches [3,4]. More recently, RNA G4s (rG4) were found in both types of ncRNAs and their dysregulation has been proposed to have an impact on disease [5]. rG4s are thermodynamically stable secondary structures in which the guanines, which are linked between themselves via Hoogsteen hydrogen bonds, engage the N1, N7, O6, and N2 atoms from each base (Figure 1) [6,7].

The stability of the negatively charged core of the G-quartet composed of O_6_ atoms, the coordination between the G-quartets, and the base—stacking interactions are governed by intercalated monovalent cations [7,8,9,10].

In case of cation absence, sequence folding towards G4 conformation is electronically unachievable, because of the negative regions of the G-quartets [10,11]. Moreover, G4’s folding and stability are directly correlated with the cation species, inherent properties and the working concentration [12]. K^+^ and Na^+^ are the most common cations used for G4 enhancement, due to their biological role and amount in cells [13]. K^+^ has a large ionic radius, which makes it able to settle in between multiple G-quartet layers; comparatively, Na^+^ is a smaller cation capable of linking up within the core of individual G-quartets [11,14,15]. Further cations, such as Li^+^ and Mg^2+^, can behave as destabilizing or neutral ions in regards to G4 formation [11].

**Figure 2:**
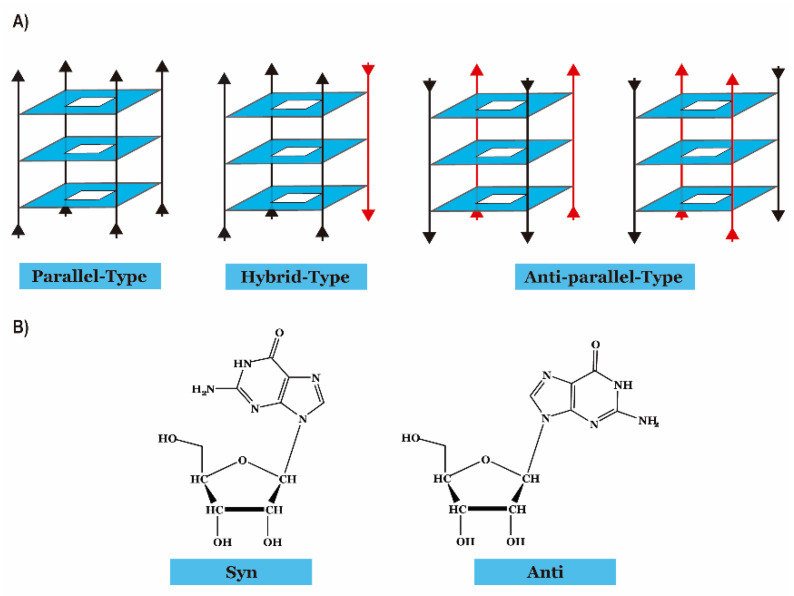
(**A**) Schematic representation of different G4 topologies (parallel, hybrid and anti-parallel). (**B**) Conformations of G4s according to strands polarity when polarity differences are associated with an angle between the G-bases and the pentose, namely to 2’ hydroxyl group.

Parallel G4 topology prevails in rG4s, wherein all strands participating in the G4 network follow the same direction (Figure 2A). Upon half the strands presenting a reverse orientation, G4 is defined as antiparallel. Once only one strand is in a contrary direction, the structure is classified as a hybrid G4 (Figure 2A) [16,17]. The 2’ hydroxyl group in rG4s generates extra interactions in comparison with DNA G4s, leading to a more stable, compact, and less hydrated profile in rG4s [18] (Figure 2B).

In addition, the folding of rG4 can also be controlled by small-molecule ligands [11,14,15,19,20], suggesting that rG4 can be druggable, being used in vivo to modulate ncRNA biological functions. Usually, the ligands’ structure is planar chromophores, which bind rG4 via π−π stacking to a terminal G-quartet, and is composed of one or more flexible substituents with a cationic charge that binds G4 grooves and loops, that is: (i) fused aromatic polycyclic systems (e.g., berberine, quarfloxin, PhenDC3, RHPS4 and BRACO-19), (ii) macrocycles (e.g., telomestatin, pyridostatin and carboxypyridostatin), and (iii) non-fused aromatic systems with flexible structural motifs.

Biophysical studies highlighted several parameters influencing rG4 conformations, including the number of G-quartet stacks, the length/sequence composition of the loops, the occurrence of bulges, the availability/nature of the central ion, the sequence in flanking regions, and the ligands interaction/stabilization of the rG4s [12].

Moreover, computational algorithms and next-generation sequencing (NGS), as well as the use of fluorescent light-up probes, have highlighted the location and biological functions of rG4s in untranslated regions (UTR) of mRNA sequences [21], and more recently in ncRNAs [22]. This was key to driving subsequent functional analyses that revealed the biological relevance of rG4s in the post-transcriptional control of gene expression impacting cellular processes. Although those computational predictions have been really helpful in proposing the importance of G4s in the transcriptome, caution should be taken, since this could lead to false positives or negatives [23]. Therefore, the use of in vitro and in vivo approaches has been growing in the last few years to probe and validate the existence of those structures.

**Figure 3:**
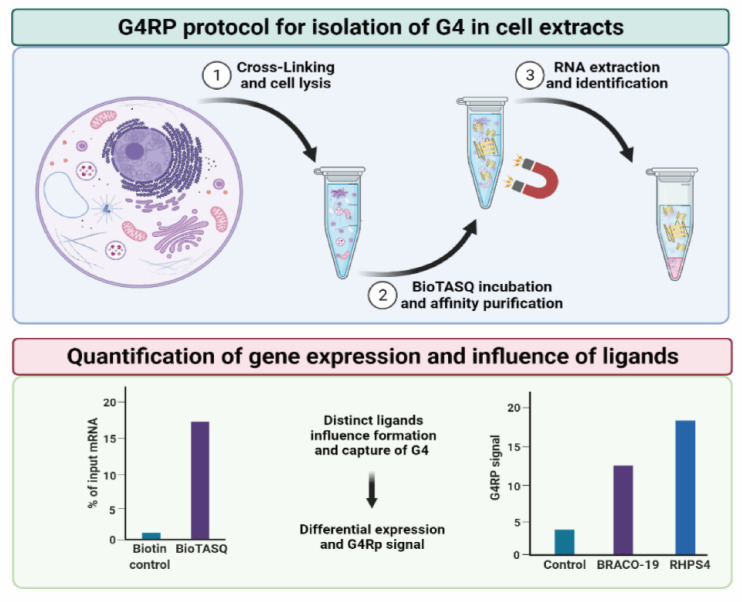
Illustration of G4RP method protocol for isolation of G4 targets from human cell extracts and the influence of the ligands in gene expression. Adapted from ref. [25]. Copyright (2018), with permission from Springer Nature.

Balasubramanian and co-workers developed an approach called rG4-seq, which is a profiling method that couples rG4-mediated reverse transcriptase stalling with NGS [24]. Firstly, the technique was employed in vitro in RNA extracted from HeLa cells, and later in vivo in bacteria and plants. Later, Monchaud and co-workers developed an approach called G4RP-seq, which combines rG4-specific precipitation (G4RP) with sequencing, to identify rG4s in human cells [25] (Figure 3). The presence of rG4s in vivo was also proved by using the G4-specific antibody BG4 and some fluorescent light-up probes, which allowed for the tracking of the folding/unfolding of G4s in living cells [26].

In those studies, it was observed that rG4s are significantly present in mRNAs (5′- and 3′-UTRs, and introns of pre-mRNAs), as well as in many classes of long and short ncRNAs, which gained significant attention in the last few years due to its ability to control several crucial biological processes, such as transcription and gene expression. An interesting review of Richter and co-workers has recently described the biological relevance and therapeutic potential of G4 structures in the human noncoding transcriptome [5].

An increasing number of reports on the presence of rG4s in ncRNAs, such as microRNAs (miRNAs) and long ncRNAs, suggest that rG4 mediated the regulation of gene expression and the control of miRNA biogenesis, opening a wide range of possibilities for new therapeutic approaches [5].

Herein we report recent findings suggesting that rG4s exist in the folded conformation of ncRNAs in living cells by using small molecules, termed rG4 small molecules ligands, which are able to modulate rG4 conformation (Table 1). Furthermore, we will explore its therapeutic potential to interfere in disease-associated ncRNAs.

In the following sections, we detail the presence of rG4s in some types of ncRNAs and their implications in the biological context.

## 2. rG4s in Telomere Long ncRNAs

rG4s have been reported in telomere-associated long ncRNAs [27,28].

Telomeres are essential to protect chromosome ends and to impose a finite lifespan to cells and tissues. Dysfunctional telomeres cause severe chromosome instability, thereby unleashing cascades of cellular reactions that are common hallmarks of cancer and premature aging [29]. Indeed, upon extensive shortening, critically short telomeres accumulate in cells and emanate an irreversible DNA damage signal, causing permanent growth arrest and, eventually, cell death. To gain unlimited replicative potential, 85–90% of human cancer cells reactivate the reverse transcriptase telomerase, which utilizes an associated RNA moiety to produce telomeric DNA [30,31]. The remaining 10–15% of human cancers elongate telomeres through homology-directed repair pathways collectively known as Alternative Lengthening of Telomeres (ALT) [32,33]. ALT can thus be considered a specialized DNA repair mechanism, assuring cell immortality. Several studies have been conducted to understand how telomeres execute their protective functions and how telomeric dysfunctions are mechanistically linked to pathological conditions. Indeed, the fact that hTERC forms rG4s structures at the 5′-terminal has several implications in telomerase activity and the function of cancer cells [28,32].

Lacroix and co-workers reported that G4 formation interferes with P1 helix formation in hTERC, a critical structural element of telomerase activity. On the other hand, DHX36, an RNA helicase member of the DEXH box family, can bind and unwind hTERC rG4s in the presence of ATP, which enables the formation of the P1 helix structure necessary for telomerase function [27]. HnRNP F/H possesses three quasi-RNA recognition motifs (qRRM) [34], which preferentially bind to poly(G)-rich sequences RNA and were reported to bind G4 sequences in 5′ tract of hTERC to regulate telomerase activity and telomere length. However, whether other RNA binding proteins exist that can associate with the 5′-end region of hTERC to regulate telomerase function is still elusive [34].

**Scheme 1:**
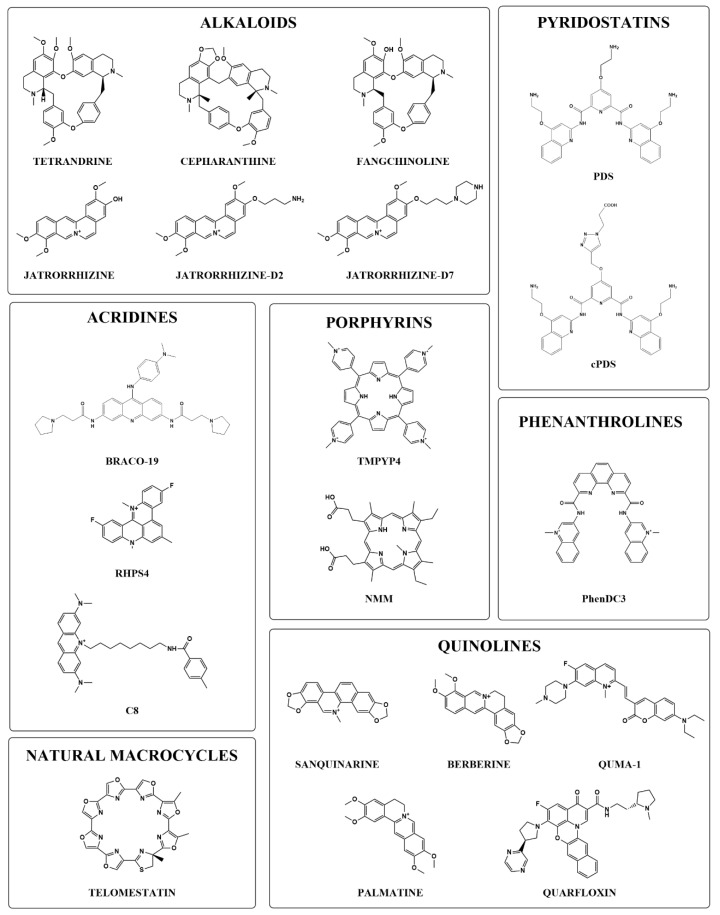
Chemical structures of the G4 ligands presented in this review. Small molecules are divided according to the chemical families. Structures were designed with ChemDraw 20.0 ^®^—PerkinElmer.

**Figure 4:**
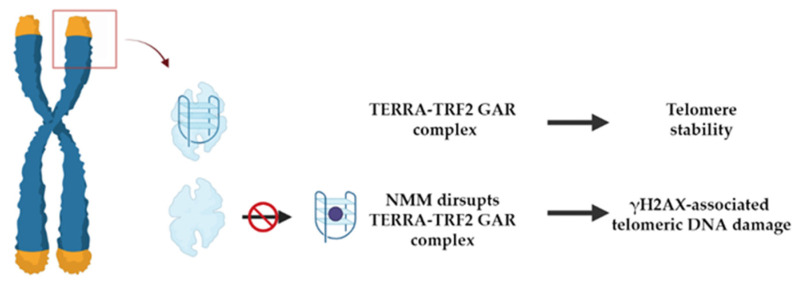
Schematic representation of TERRA rG4 interaction with TRF2 GAR domain that is required for telomere stability and integrity. Treatment of human melanoma cells with mesoporphyrin IX (NMM) leads to disruption of TERRA and the induction of γH2AX-associated telomeric DNA damage.

Another telomere-associated lncRNA is the telomeric repeat-containing RNA (TERRA), which was also reported to form rG4s and be involved in the regulation of telomerase activity and DNA telomere length [35]. TERRA RNA is localized to chromosome ends in the nucleus, suggesting a link between TERRA rG4s and telomere function [36]. Several biological functions have been associated with TERRA tandem repeats, namely, modulation of heterochromatin regulation, telomerase inhibition, telomere length regulation, and telomere protection. For instance, TERRA rG4s can interact with telomeric DNA G4s to form intermolecular hybrid G4 structures and suppress telomerase activity [35]. A study of Balasubramanian and co-workers showed that TERRA recruits TRF2, an important protein that regulates the association of TERRA and telomeric DNA [37]. Recently, a study developed by Lieberman and co-workers reported that the G4 structure of TERRA is a recognition element for the TRF2 GAR domain [37,38]. They reported that the interaction between TERRA and TRF2 GAR domain is of utmost importance to maintain telomere stability and regulation. In their study, they used the well-known G4 ligand N-methyl mesoporphyrin IX (NMM: represented in Scheme 1) to disrupt the TERRA-TRF2 GAR complex [39]. Only NMM showed preferential binding to TERRA. The results showed the loss of TERRA, and the induction of γH2AX-associated telomeric DNA damage associated with decreased telomere length, and increased telomere aberrations (Figure 4) [39].

## 3. rG4s in Pri-miRNAs

miRNAs are produced by a highly coordinated series of enzymatic cleavages from pri-miRNA to its mature form miRNA via its premature form (pre-miRNA) [40]. There are a few reports which have described G4s in pri-miRNAs compared to those found in pre-miRNAs, which have paved the way to improving the knowledge on pri-miRNA processing and the outcome in the miRNA biogenesis pathway [5].

Perreault et al. identified G4 located near the Drosha cleavage site in three distinct pri-miRNAs (pri-mir200c, pri-mir451a, and pri-mir497) by Reverse Transcription Stalling (RTS) [41]. The G4 folding in pri-miRNAs was unstable, being detected by in vitro methods only in the presence of ligands cPDS and PhenDC3 (Scheme 1). Nevertheless, the authors showed that mutations disrupting G4 folding had an impact on the processing of the pri-miRNAs, leading to low levels of miRNAs. These were explained by the dynamic shifts between G4 and hairpin folding, which influences the pri-miRNA processing [41]. Considering the tumor suppressor nature of the mentioned pri-miRNAs, this approach could be further explored to develop novel anticancer therapies. Moreover, Perreault et al. showed that binding small antisense oligonucleotides to the pri-miRNA can modulate mature miRNA levels [41]. Considering G4’s impact on pri-miRNA, a processing analysis of potassium ion dependence could be the best starting point when identifying and validating G4 formation in pri-miRNAs.

**Figure 5:**
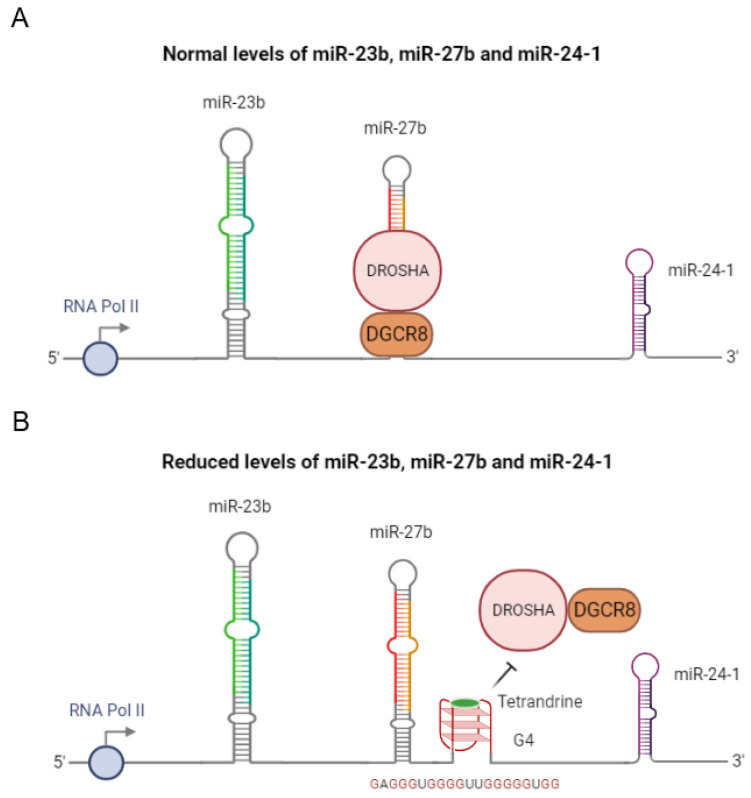
Representation of the influence of G-rich sequence in normal (**A**) and reduced (**B**) levels of miR-23b, miR 27b and miR-24-1. Adapted from ref. [42]. Copyright (2021), with permission from Oxford University Press.

A recent report by Ming Xu showed that G4 formation in vitro and cells prevented Drosha-DGCR8 binding and processing of the pri-miR, suppressing the biogenesis of the three miRs (miR-23b/27b/24-1) which (Figure 5A) are related to cardiac disease [42]. The formation of rG4 could negatively regulate the production of the miRNAs encoded by the cluster and, in the presence of tetrandrine, a significant reduction of all three miRNAs occurs. Disruption of this intragenic G4 in these pri-miRs increased the production of all three miRs. Interestingly, tetrandrine also affects the expression levels of pre-miRNAs instead of pri-miRNAs, suggesting that its mechanism of action in miRNA biogenesis takes place during the generation of pre-miRNA from pri-miRNA [42] (Figure 5B). Conversely, the binding of ligand tetrandrine to G4 stabilized the structure and suppressed miRs production in human and rodent cardiomyocytes (Figure 5 and Scheme 1) [42].

However, to date, these ligands did not recognize just one G4; and for these reasons, finding molecules specific for G4 in the pri-miRNA structure may prove more difficult, but the field is underexplored, and thus applications such as microarrays or high-throughput screening methods towards different rG4 structures may provide new insights for G4 in pri-miRNAs.

## 4. rG4s in Pre-miRNAs

In the last few years, the presence of G4 structures in pre-miRNAs has been studied more, comparing their presence to those found in pri-miRNAs. In 2015, bioinformatics predictive tools, using a GQRS mapper [43], suggested the presence of at least one rG4 motif in 16% of human pre-miRNA stem−loop regions [44]. Recently, Perreault and co-workers, using a new approach, reported that only 2% of pre-miRNA contain a putative rG4 motif [22]. This analysis confirms a competition between G4, and the secondary structures recognized by Dicer identifying that rG4 overlapping the Dicer cleavage site in 9% of the miRNAs. In this sense, several studies have reported the presence of the equilibrium between G4 and stem−loop in pre-miRNA and its effect in the biological process [22].

**Figure 6:**
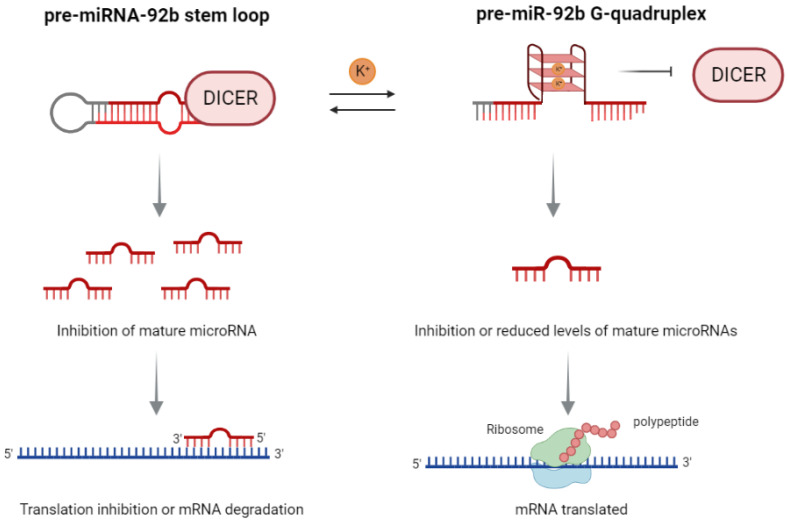
Representation of the ionic profiling to induce G4 formation by unwinding the stem−loop of pre-miR-92b Adapted from [44] copyright (2015), with permission from Elsevier.

Arachchilage et al. reported the presence of a very stable G4 structure in the human pre-miR-92b [44]. The human miR-92b is a clinically important miRNA and is significantly upregulated in several human cancers [45,46,47,48]. The G4 structure of pre-miR-92b is very stable and has six G stretches, each containing three G4. Using several biochemical and biophysics approaches, the G4 motif in pre-miR-92b was proven in cytoplasmatic K^+^ concentrations (Figure 6). The presence of the rG4 structure exists in equilibrium with the canonical stem−loop structure, and this equilibrium regulates the maturation of pre-miR-92b [44]. In this sense, a rational design of locked nucleic acid (LNA) able to bind specifically the rG4 motif conformation of pre-miR-92b was carried out [49]. The LNA treatment demonstrated the importance of increasing PTEN levels in NSCLC through suppression by miR-92b maturation [49].

Cruz and co-workers used a labeled pre-miR-92b sequence as a molecular recognition probe of a microfluidic platform for the recognition of the nucleolin (NCL), an important biomarker, in biological samples. The G4 motif present in pre-miR-92b was used for the molecular detection of NCL. The additional stability of the rG4 structure was provided by ligand C_8_ (Scheme 1) [50]. C_8_ is an acridine orange derivative [51] that has been reported as a promising G4 binder [52,53,54,55].

Additionally, Panday et al. used the pre-miR-let-7e to study the equilibrium between G4 structure and stem−loop in RNA in a more biologically relevant environment [56]. Pre-miR-let-7e contains a G2 with a total loop length of seven nucleotides, and the Dicer cleavage assay suggests a role for the G4 structure at physiologically relevant concentrations of Mg^2+^ and K^+^, leading to the reduction of miR-let-7e levels. To study the importance of the potassium-dependent G4 structure, cells were treated with TMPyP4, leading to an increase in miRNA levels. It is probable that TMPyP4 promotes the disruption of G4 and shifts the equilibrium between G4 and the stem−loop [56].

**Figure 7:**
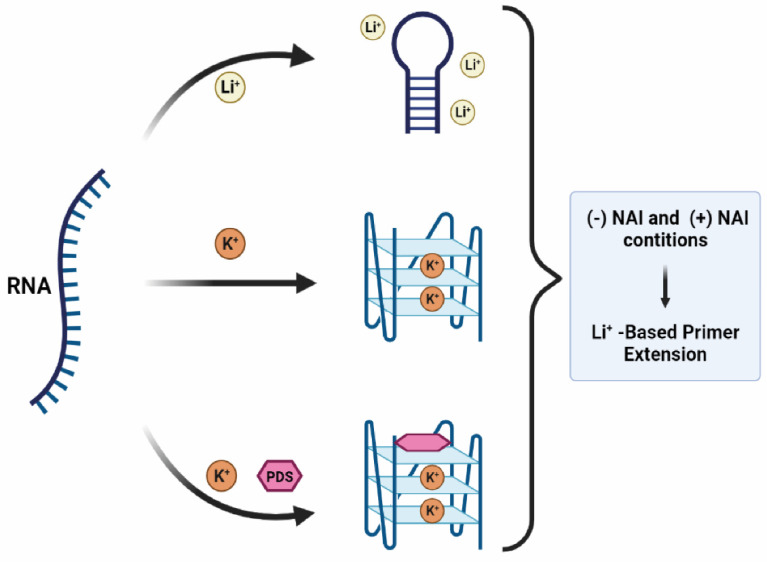
Representation of SHALiPE method [57]. Copyright (2016), with permission from Wiley.

Balasubramanian and co-workers used a new approach to map the G4 formation in RNAs. This new method, called SHALiPE, couples selective 2′-hydroxyl acylation with a lithium ion-based primer extension, and recognizes the structural characteristics for rG4 mapping. They used SHALiPE to prove the formation of an rG4 structure in pre-miR-149. Additional biophysics studies support the SHALiPE and have proven that rG4 has a parallel topology with high thermal stabilization under physiological K^+^ conditions (Figure 7). The authors still used the G4 ligand and pyridostatin (PDS) (Scheme 1), and the results provided strong evidence that pre-miR-149 can form G4 structure in the presence of K^+^ and PDS. The presence and stabilization of the G4 structure inhibits the Dicer processing activity in vitro and decreases the miR-149 production [57].

Additionally, Cruz and co-workers studied the thermal stabilization of the pre-miR-149 G4 in the presence of two acridine orange derivatives, C_8_ and C_8_-NH_2_ (Scheme 1) [58]. The results showed that the G4 structure can be stabilized by the C_8_ derivative with more effectiveness than C_8_-NH_2_. These results were anticipated by molecular dynamics in an in silico study [59]. Ghosh et al. used the porphyrin TMPyP4 (Scheme 1) to destabilize the G4 structure formed from the pre-miR-149. Biophysics studies, such as CD, UV-melting, ITC and NMR showed that TMPyP4 binds strongly to G4 and unfolds it, and upon addition of TMPyP4, the dynamic change occurs to the hairpin structure, enhancing the transcript levels of mature miR-149 in cells [60].

Mihailescu and co-workers reported the presence of a parallel intramolecular G4 structure with six G-tracts in pre-miR-1229. They used several biophysical techniques to show an equilibrium between the G4 structure and the canonical hairpin. G4 structure mature miR-1229 has been shown to directly control the expression of SORL1, one protein responsible for the processing and trafficking of β-amyloid proteins [61]. Whilst the G4 structure within pre-miR-1229 has not been stabilized by any ligand, it could potentially become a therapeutic target in Alzheimer’s disease, through ligands that are able to stabilize the G4 and consequently reduce the levels of mature miR-1229. Additionally, the G4 forming motif could be an important therapeutic target in other pathologies such as colorectal [62] and breast cancer [63].

Similarly, miR-26a has been described in several physiological processes, is emerging as a therapeutic target for human diseases, and generally functions as a tumor suppressor [64,65]. Fu and co-workers identify a guanine-rich sequence in pre-miR-26a-1 that can fold into the metastable G4 structure containing 2-quartet in vitro and in vivo [66]. This sequence could regulate miR-26a function and expression levels. Using one helicase (DHX36), they are able to bind and unwind the G4, promoting miR-26a maturation. Moreover, the treatment of cancer cell lines with PDS and carboxy pyridostatin (cPDS) (Scheme 1) significantly decreases miR-26a levels, suggesting that G4 structure inhibits endogenous miR-26a [66].

Recently, Cruz and co-workers reported the formation of G4 structure in the human pre-miR-150 [67]. Circular dichroism studies indicated the formation of a parallel G4 in the presence of K^+^ and PhenDC3 (Scheme 1). Biophysics studies have shown that the pre-miR150 G4-forming sequence recognizes and interacts with the NCL protein. This sequence still shows co-localization with NCL in a lung cancer cell line and in peripheral blood mononuclear cells [67]. Considering that human miR-150 plays an important role in several cancer developments [68], the G4-forming region found in precursor pre-miR-150 can be an attractive therapeutic target.

## 5. rG4s in miRNAs

**Figure 8:**
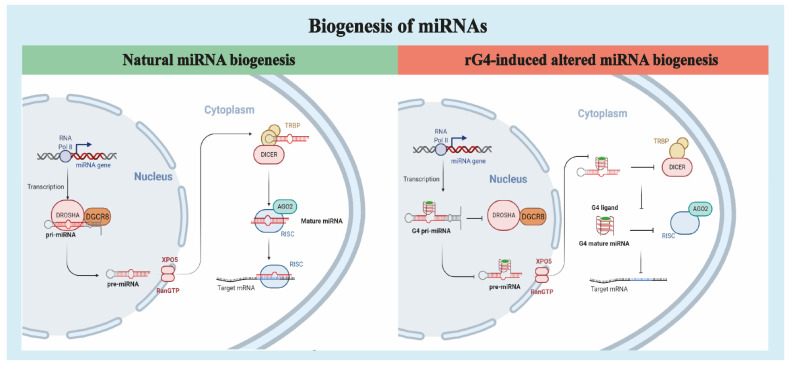
Biogenesis of miRNAs. Comparison of natural biogenesis versus interference of the rG4s in the biogenesis and function of miRNAs.

rG4s play roles in every step of the miRNA biogenesis and function (Figure 8) [5]. Pri-mRNA rG4s regulate Drosha-mediated processing, pre-miRNAs rG4s inhibit DICER-mediated maturation, and miRNA rG4s abolish its loading onto RISC (Figure 8) [69,70,71]. The understanding of the mechanisms that control miRNA biogenesis may allow for the development of tools to modulate the expression of specific miRNAs, which is crucial for the development of novel therapies for human disorders derived from the aberrant expression of miRNAs.

Several studies have demonstrated the in vitro formation of rG4s in miRNAs and the competition with the RNA stem−loop conformation, which have crucial roles in post-transcriptional regulation [27,70,71]. Indeed, these studies revealed the important role that rG4 structures have in impairing miRNA−mRNA interaction [72]. However, the current structural information and cellular roles of the rG4s in miRNAs remain elusive [73]. The latest release of miRBase (version 22) deposited 38,589 hairpin precursors and 48,860 mature microRNAs from 271 organisms [74]. So far, by bioinformatics analysis, 166 rG4-positive human miRNAs sequences (6%) using two different G4 prediction tools (G4-detection algorithm G4NN and the Quadparser-based (G_2+_N_1–12_)_3_G_2_) were identified [75]. In the last few years, the miRNA rG4 formation, and targeting by different types of G4 ligands, revealed a new possible mechanism for regulating microRNA functions with therapeutic potential.

The first research relating the rG4 in miRNA and ligands was published in 2015 by Ming Xu and collaborators [76]. This study evaluated the formation of G4 in the genomic cluster near three distinct microRNAs, namely, miR-23b, miR-27b, and miR-24 (cluster miR-23b-27b-24-1). The miR clusters can display tumor-suppressive or oncogenic roles depending on their context, as well as the individual members of the cluster that are able to adopt suppressor or oncogenic promoter behaviors according to the cancer type [77]. The authors found three potential G4 sequences capable of folding in antiparallel, and one in parallel, conformations, and tested six small molecules (tetrandrine, fangchinoline, palmatine, jatrorrhizine, berberine and cepharanthine; Scheme 1) [76]. Surprisingly, some of the tested ligands (tetrandrine, fangchinoline, and cepharanthine) evidenced selective binding affinity to parallel rG4 instead of antiparallel conformations. This effect can be explained due to the unusual chemical structure (non-planar molecules) of these natural alkaloids, which allows for binding via locating on the groove formed by the loops [78].

**Figure 9:**
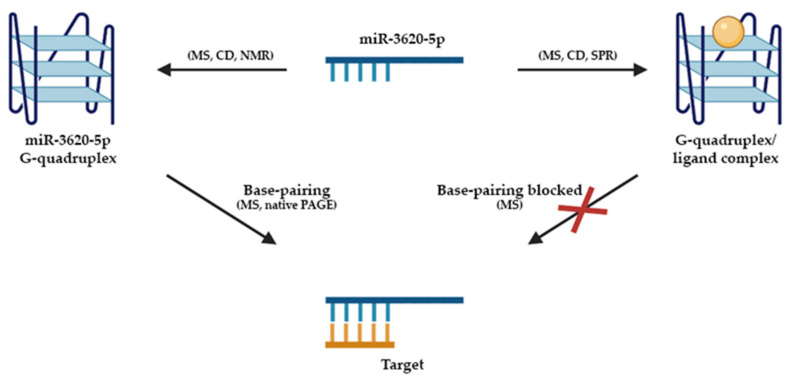
Representation of G4 formation in miR-3620-5p and the inhibition effect of sanguinarine on the base pair formation of miR-3620-5p with its target sequence. Adapted from ref. [73]. Copyright (2016), with permission from Elsevier.

Yuan et al. published a second study reporting the formation and folding of a stable parallel rG4 in miR-3620-5p in physiological conditions by using a set of biophysical techniques such as ESI-MS, CD, NMR, and SPR [73] (Figure 9). This miR is reported to be associated with different kinds of cancers, such as gastric cancer and metastasis [79]. Additionally, the same authors concluded that sanguinarine, a natural alkaloid used in traditional Chinese medicine, binds and stabilizes the rG4 in miR-3620-5p [73]. Furthermore, the study revealed that this interaction among rG4 and sanguinarine can block the base pairing between miR-3620-5p and its target sequence, proposing a mechanism to regulate miRNA function, affecting the miRNA−mRNA interactions through the miRNA rG4 formation and targeting by ligands [73].

Later, the same research group also proved the formation of the rG4 miRNA-1587 [80,81]. This miRNA is responsible for the proliferation, migration and tumorigenicity of cells in glioma and breast cancer [82,83]. It is noteworthy that miR-1587 formed a dimeric G4 through the 3′-to-3′ stacking of two monomeric G4 subunits with one NH_4_^+^ sandwiched between the interfaces [80]. The dimeric rG4 was also adopted under molecular crowding conditions or in the presence of a jatrorrhizine ligand [80]. Another study conducted by Yuan and collaborators, which focused on miR-1587, showed that the formation of a secondary structure affects the ability of miR-1587 to bind the target mRNA sequence and, consequently, reduces its expression levels [81]. This conclusion shows the potential for therapeutic applications. Moreover, the pseudopalmatine ligand prevents an interaction with the target gene via G4 stabilization and has the same effect on the suppression and modulation of gene expression. Contrarily, TMPyP4 revealed the disruption of rG4 promoting the binding of miR15-87 to its target, allowing for the mRNA processing [81]. In addition, through bioinformatic analysis, the authors identified other miRNAs able to form rG4, namely, miR-197-5p, miR-765, miR-3620-5p, and miR-5196-5p [81].

Using the G4NN algorithm, specially developed to detect RNA G4s, Kwok and co-workers identified up to 478 putative RNA G4 human miRNAs [76]. However, when they increased the stringency by using Quadparser, only 166 human miRNAs were positive for the presence of a putative rG4 structure. From those 166 human miRNAs, they selected four miRNAs (miR-149, miR-197, miR-432 and miR-765), taking into account the biological relevance, conservation, as well as confidence of G4 formation based on G4NN parameters. It is noteworthy that these miRNAs have been linked to several types of cancer. For instance, dysregulation of miRNA-149 has been implicated in cancer migration and invasion [84]. Therefore, their study provides a strong basis for the involvement of RNA G4s in post-transcriptional regulation and paves the way for future investigations [75]. Moreover, they showed that the NMM ligand stabilizes in vitro rG4 of these four miRNAs in a specific manner, modulating gene expression [75].

Zhou et al. identified and characterized rG4 in miR-92a by ESI-MS and CD [85], which is associated with retinoblastoma, cervical and non-small cell lung cancers [86,87,88]. The study evidenced the formation of a parallel rG4 in distinct ionic conditions and adopts a dimeric structure in the presence of NH_4_^+^. Moreover, the palmatine ligand has a high binding affinity and stabilization effect (increasing its thermal stability by 10 °C) on the dimeric rG4 in miR-92a [85].

Recently, Vivekanandan’s et al. performed an analysis of G4 upstream of herpesvirus miRNAs, namely in Kaposi’s sarcoma-associated Herpesvirus miR-K12–1-9,11 cluster and Human Cytomegalovirus miR-US33 [89]. They also characterized a parallel rG4 in these miRNAs and showed that TMPyP4 and PDS interfered with rG4 stabilization modulating the promoter activities of KSHV miR-K12 and HCMV miR-US33.

Other examples of miRNA rG4s have also been reported [75,69]. A list with the G4 sequences in ncRNA nucleic acids discussed in this review, as well as their ligands, biological target, and effect of the G4 formation is highlighted in Table 1. Overall, these results proved the influence of rG4s in miRNA biogenesis and consequently in several important biological processes such as regulation of gene expression and transcription.

## 6. Conclusions

The bioinformatics tools showed that most of the human transcriptome is composed of ncRNAs with the widespread occurrence of potential G4-forming sequences. The representative roles of ncRNAs rG4 on a myriad of biological processes in cells needs to continue to be verified to illustrate their utility in association with diseases. The potential for exploiting rG4 small molecules to control miRNA-regulated target gene expression based on high-order rG4 structures must be exploited in vitro and in vivo to modulate ncRNA biological functions.

Overall, the developments described in this review showed that the research on ncRNA G4s needs to be protracted, and the transient nature of the rG4 structures at the cellular level, which are quite unstable, can be surpassed by the presence of small molecules. Indeed, multiple overlapping structures can exist within the same RNA molecule, and these structures may undergo equilibrium shifts as per the cellular requirement. In this context, the use of small molecules that selectively recognize G4s over other structures may prove effective. Their regulatory role over ncRNA targets and the effect on resultant protein products remain to be elucidated. However, the research on rG4s is expanding, and new rG4 small molecules with new functions at the cellular level will be available, allowing the development of novel therapeutic approaches in pathological rG4 ncRNAs-linked contexts.

## Figures and Tables

**Table 1:** List of G4 sequences in ncRNA nucleic acids discussed in this review.

		rG4 Forming Sequence (5′-3′)	Ligands	Biological Target	Effect of G4 Formation	Refs.
Gene Cluster	mir-23b	GAGGGTGGGGGGTTGGGGGTGG	Tetrandrine	Drosha-DGCR8	Decreased of pri-miR levels	[42]
	mir-27b					
	mir-24-1					
	miR-K12–1-9	GGGTGGGAGGAAGGATGTGGGGGTGGG	TmPyP_4_, PDS	promoter activity	Transcriptional regulation of virus encode miRNA.	[89]
Pri-miRNA	Pri-mir200c	GGUGGGCGGGCUGGGCGGGGG	cPDS, PhenDC3	Drosha-DGCR8	Reverse Transcription Stalling	[41]
	Pri-mir451a	GGGCACUUGGGAAUGGCAAGG				
	Pri-mir497	GGAGGGGGUGGG				
Pre-miRNA	pre-miR-92b	GGGCGGGCGGGAGGG	LNA, C_8_	Dicer	Decrease mature levels of miRNA	[49,50]
	pre-miR-let-7e		TmPyP_4_		Increase mature levels of miRNA	[56]
	pre-miR-149	GGGAGGGAGGGACGGGGG	PDS, C_8_		Decrease mature levels of miRNA	[57,58]
	pre-miR-1229	GGGUAGGGUUUGGGGGAG AGCGUGGGCUGGGGUUCAGGG ACA		Dicer		[61]
	pre-miR-26a-1	GGAUAGGCUGUGCAGGUCCCAAUGGG	PDS, cPDS		Decrease mature levels of miRNA	[66]
	pre-miR-150	GGCCUGGGGGACAGGGACCUGGG	PhenDC3			[67]
miRNA	miR-3620-5p	GUGGGCUGGGCUGGGCUGGGCC	Sanguinarine	Blocked the base pairing with target sequence	Depression of miRNAs	[73]
	miRNA-1587	UUGGGCUGGGCUGGGUUGGG	Jatrorrhizine derivatives, pseudopalmatine			[80,81]
	miRNA-149	AGGGAGGGACGGGGGCUGUGC	NMM			[75]
	miRNA-197	CGGGUAGAGAGGGCAGUGGGAGG				
	miRNA-432	UCUUGGAGUAGGUCAUUGGGUGG				
	miRNA-765	UGGAGGAGAAGGAAGGUGAUG				
	miR-92a	TAATGGGGTGGGGGCTGGGAA	Palmatine			[85]

## Data Availability

Data is contained within the article.
